# Challenges of Pharyngeal Cancer Screening in Lower-Income Countries during Economic and Social Transitions: A Population-Based Analysis

**DOI:** 10.3390/ejihpe13100157

**Published:** 2023-10-10

**Authors:** Andreea M. Kis, Claudia G. Watz, Alexandru C. Motofelea, Sorin Chiriac, Marioara Poenaru, Cristina A. Dehelean, Claudia Borza, Ioana Ionita

**Affiliations:** 1 Department of ENT, Faculty of Medicine, “Victor Babeş” University of Medicine and Pharmacy, 2nd Eftimie Murgu Sq., No. 2, 300041 Timișoara, Romania; kis.andreea@umft.ro (A.M.K.); marioara.poenaru@gmail.com (M.P.); 2 Department of Pharmaceutical Physics, Faculty of Pharmacy, “Victor Babeș” University of Medicine and Pharmacy, Eftimie Murgu Sq., No. 2, 300041 Timișoara, Romania; 3 Research Center for Pharmaco-Toxicological Evaluations, Faculty of Pharmacy, “Victor Babes” University of Medicine and Pharmacy, Eftimie Murgu Sq., No. 2, 300041 Timisoara, Romania; cadehelean@umft.ro; 4 Department of Internal Medicine, Faculty of Medicine, “Victor Babeş” University of Medicine and Pharmacy, Eftimie Murgu Sq., No. 2, 300041 Timișoara, Romania; alexandru.motofelea@umft.ro; 5 Department of General Surgery, Faculty of Medicine, “Victor Babeș” University of Medicine and Pharmacy, Eftimie Murgu Sq. No. 2, 300041 Timișoara, Romania; 6 Department of Toxicology, Faculty of Pharmacy, “Victor Babeș” University of Medicine and Pharmacy, Eftimie Murgu Sq., No. 2, 300041 Timișoara, Romania; 7 Department of Physiopathology, Faculty of Medicine, “Victor Babeș” University of Medicine and Pharmacy, Eftimie Murgu Sq., No. 2, 300041 Timișoara, Romania; borza.claudia@umft.ro; 8 Department of Hematology, Faculty of Medicine, “Victor Babeş” University of Medicine and Pharmacy, Eftimie Murgu Sq., No. 2, 300041 Timișoara, Romania; mdioanaionita@yahoo.com; 9 Multidisciplinare Research Center for Malignant Hemopathies (CMCHM), “Victor Babes” University of Medicine and Pharmacy, Sq., No. 2, 300041 Timișoara, Romania

**Keywords:** pharyngeal cancer, HNC, screening, adenopathy stage (N0/N1)

## Abstract

*Background and Objectives:* The rate of head and neck cancer (HNC) is expected to increase by 30% by 2030. However, there are many similarities between the symptomatology of a benign and a malign diagnosis; thus, a protocol for conducting a full head and neck examination is of high importance since the absence of adenopathy does not exclude a malignant diagnosis and also a favorable prognosis. *Material and methods:* The current study presents a retrospective study on 515 adult patients who underwent a biopsy for possible head and neck tumor pathology. Results: The patients identified with cancer were older than the rest of the group, with a higher developing trend in men than in women. However, the top 10 symptomatology patterns were identical in the malign and benign groups, meaning that new HNC may be missed due to the common symptomatology between benign and malign outcomes. *Conclusions:* The importance of a full ear, nose, and throat (ENT) examination may be of significant relevance for a proper diagnosis that can improve the overall prognosis of a patient with cancer. The absence of routine screening tests and screening guidelines for oral and pharyngeal cancers represents a significant barrier to secondary HNC prevention.

## 1. Introduction

Each year, more than 500,000 new diagnoses of head and neck squamous cell carcinoma (HNSCC) are reported worldwide [1]. The incidence of HNC is increasing, with an expected rise of 30% by 2030, equivalent to a total of 1.08 million new cases annually. It is also important to highlight that men are generally more prone (2–4 times more likely) to develop HNC than women [2]. The risk factors consist of several aspects: tobacco smoking, alcohol consumption, and exposure to various environmental pollutants and different infections from viral agents, such as (i) human papillomavirus (most commonly HPV-16, followed by HPV-18 and other strains) and (ii) Epstein–Bar virus, known as an etiologic risk factor for the nasopharynx [3,4]. Tobacco consumption and alcohol intake are two aspects responsible for approximately 75% of the cancers related to the lip, oral cavity, and pharynx in Western Europe [5,6]. Aging, precarious oral hygiene, and low-vegetable diets are also part of the risk factors [7,8]. Nevertheless, the survival rate for HNC has improved modestly in the last 30 years; the incidence rate in males exceeds 20 per 100,000 in several regions, including Hong Kong, the Indian subcontinent, different countries in Europe, and Brazil [9]. For example, the Surveillance, Epidemiology, and End Results (SEER) registry revealed that the 5-year survival rate changed from 55% (1992–1996) to 66% (2002–2006) when taking into account all age groups and anatomical sites. Nevertheless, when referring to developing countries, Romania, which, according to an economic and social situation study, situates itself within the lower-income-countries group [10], presents a high annual incidence of 2388 new cases (Table 1) based on the estimated age-standardized rates of both men and women developing HNC [9,11].

Around 30–40% of pharyngeal cancer patients are diagnosed with early stage disease and have a 5-year survival rate of 70–90% when under treatment. However, the majority of pharyngeal cancers are discovered at advanced stages (over 75%), when medical treatment is not so efficacious, and surgical treatment deteriorates the organs involved in the speech and swallowing processes [12]. Cervical positive lymph nodes are considered key prognostic factors in HNC and are closely related to recurrence. The survival rate for patients who live in countries with limited access to tertiary healthcare opportunities is very low, showing a rate of around 30–40% [5]. Hypopharyngeal carcinomas often emerge at a late stage (stage 3 or stage 4), making curing them less likely than curing other cancers. There are many critical prognostic markers for carcinoma of the hypopharynx, including the architecture and location of the tumor [13]. Patients diagnosed with HNC (advanced stage) with no clinical history of a pre-malignancy in the oral cavity are subjected to surgical resection, followed by radiotherapy (accompanied or not by chemotherapy), based on the disease stage. Chemotherapy mainly comprises several agents, such as cisplatin, paclitaxel, docetaxel, 5-fluorouracil, methotrexate, and cetuximab [14].

However, advanced-stage chemoradiotherapy (CRT) is the primary approach for treating cancers developing in the pharynx. Nevertheless, HPV-positive HNC generally presents a more favorable prognosis when compared to HPV-negative HNC. Still, several studies are investigating the relationship between dose reduction (of both radiation and chemotherapy) and HPV-positive disease efficacy. According to Bonner et al., most patients diagnosed with HNC need multilateral approaches and, thus, multidisciplinary care [13]. A delay in diagnosis and treatment leads to increased tumor stages and could affect local tumor control and patient survival. Recent guidelines of the French Society of ENT (SFORL) state that patients should be treated within 30 days of their first appointment [15]. Access to services depends on a referral from primary care physicians or immediate access in an emergency (through the emergency room). Once a diagnosis has been made, all specialized services (such as surgery, cytostatics, and/or radiotherapy) should be immediately accessible.

Taking into account all the aspects presented above related to HNC diagnosis and prognosis, the present study aims to compare the diagnostic strategies employed by practitioners and the relevance in the relapse rate and survival based on the initial stage of lymphadenopathy (N0/N1) of the patients upon admission to the hospital.

## 2. Materials and Methods

### 2.1. Study Design

The present study represents a retrospective cohort study of 515 patients older than 18 years who underwent a biopsy for a possible head and neck tumor pathology. The study focused on patients’ malignant histopathological diagnosis, including several aspects, such as date of diagnosis, date of death, treatment of head and neck cancer, classification of histological diagnosis at three levels: pharynx, adjuvants, and anti-neoplastic therapies. The patients were carefully selected from Info World-Hospital Manager between January 2014 and December 2018 using the diagnosis-related group codes: malignant tumor; lesion exceeding the oropharynx (C.10.8); unspecified nasopharyngeal malignant tumor (C11.9); malignant tumor of the piriform sinus (C12); unspecified hypopharyngeal malignant tumor (C13.9); tumor of the lip, oral cavity, and pharynx with unpredictable and unknown evolution (D37.0); unspecified tumor with unpredictable and unknown evolution (D48.9) at Timisoara Municipal Emergency Clinical Hospital, Department of Otorhinolaryngology.

### 2.2. Data Collection

The data recorded were initial diagnostic tumor pathology with unpredictable evolution, benign, or malignant biopsy related to the diagnosis. However, the focus was on the group of patients with malignant disease, and the following aspects were evaluated: age at diagnosis, gender, rural or urban environment, localization of the tumor (nasopharynx, oropharynx, hypopharynx). TNM staging was determined retrospectively, where possible, using disease extent and collaborative staging codes for tumor sizes and locations, following the classification protocol developed by the American Joint Committee on Cancer: days of hospitalization, type of hospitalization (emergency or not), type of surgical intervention, type of biopsy, histological diagnosis, clinical presentation, and overall survival (OS). The study examined patients with malignant head and neck tumors. Data collected included date of diagnosis, date of death, treatment details, and histopathological classification. The tumors were classified via anatomical location in the pharynx. Information on adjuvant therapies and anti-cancer treatments administered was also collected.

The primary outcome was defined as the time (in months) from diagnosis to death from any cause, whereas for OS, the time was recorded from the moment of diagnosis to cancer-related death.

Exclusion criteria consisted of patients under age of 18 years, without histopathological diagnosis or non-concluded biopsy and laryngeal carcinoma. Also, patients with incomplete staging, treatment, or follow-up data were excluded, or with other anatomic site codes (nasal cavity and paranasal sinuses, salivary glands, thyroid, and unknown primary site).

### 2.3. Statistical Analysis

Data were collected and analyzed with R (version 3.6.3) using Tidyverse, Final-Fit, MCGV, Survival, Stringdist, Janitor, and Hmisc packages. Results were presented as mean and standard deviation for continuous variables with Gaussian distribution, medians and IQR range for continuous variables with no Gaussian distribution. Frequency and percentages were used for categorical variables. To assess the significance of differences between groups, Students’ *t*-test or analysis of variance (means, Gaussian populations) were used for normal distributed data and Mann–Whitney U-test or Kruskal–Wallis tests were reported (medians, non-Gaussian populations). Continuous variable distributions were tested for normality using the Shapiro–Wilk test and for equal variance using the Levenes test. The strength of association between two continuous variables from non-Gaussian populations was assessed using Spearman’s correlation coefficient. A sample size calculation was performed prior to the study, with the aim of achieving a 95% confidence level and at least 80% statistical power. In this study, *p* < 0.05 was used as the threshold for statistical significance.

### 2.4. Ethical Considerations and Approval

The current study was evaluated and approved by the Scientific Research Ethics Committee of Victor Babeș University of Medicine and Pharmacy, Timișoara (Approval No. Nr. 53/28.09.2018 (2023)).

## 3. Results

A total of 515 of patients were included in this study with a possible pharyngeal tumor who underwent surgical treatment following diagnosis during the study period. The median age at diagnosis was 55.7 years, with a significant difference in gender distribution: 125 females (24.3%) and 390 males (75.7%).

Data showed that patients with cancer were older, with a mean age of 59.7 (9.8) years versus 47.3 (15.6) years in the benign group (*p* = 0.001). Also, the urban population was higher with 181.0 (51.7%) malign patients versus 169 (48.3%) patients from rural environments.

Of the 515 patients with possible malignant tumors, 165 patients had a benign histological diagnosis and 350 patients presented a malignant diagnosis during this time period, limited to the pharynx. Regarding the tumor distribution of the pharyngeal subsites, in the benign group, 62.4% were located in nasopharynx and 33% within the oropharynx compared to the malign group, where the hypopharynx was responsible for the tumor of 42% of the patients, followed by the oropharynx with 41.1%. For nodal staging, the following distribution was observed: 345 (67%) of the total number of patients (513) presented with N0, and 54.0 (15.4%) presented with N1 staging from the malign group. A total of 199 (56.9%) had a malign biopsy, while 146 were benign. Among the 350 patients diagnosed with malignancy, 61 (1.4%) patients presented with N3, 54.0 (15.4%) patients were diagnosed with N1, and 36.0 (10.3%) presented with N2. Hospitalization days ranged from 1 to 37 days, with a mean of 4.9, influenced by emergency enrolment. All results obtained are summarized in Table 2.

### 3.1. Clinical Presentation

**Figure 1:**
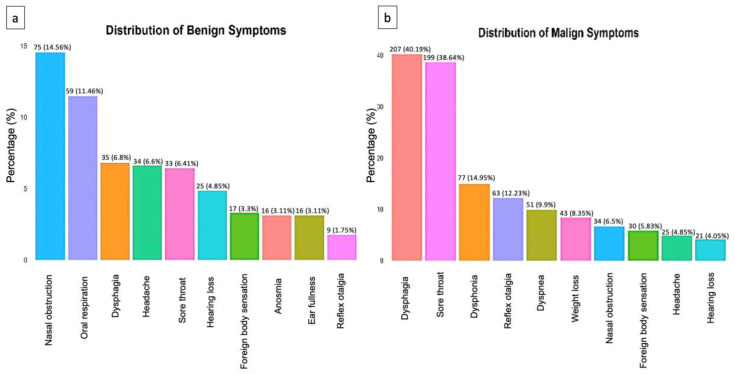
(**a**) Distribution of the top 10 non-malignant symptoms. (**b**) Distribution of the top 10 malignant symptoms.

A total of 38 different symptoms were reported in patients with benign and malignant tumors. Additionally, 10 symptoms were common to both groups, but with different frequencies. In the malignant tumor group, the most common symptoms were dysphagia (40%), sore throat (39%), dysphonia (15%), and reflex otalgia (12%) (Figure 1a).

In the group with benign tumors, the most common symptoms were nasal obstruction (15%), oral respiration (11%), and dysphagia (7%) (Figure 1b).

However, because benign and malignant symptoms overlap, clinical investigation must be performed thoroughly and a follow-up employed at all times to accurately rule out a possible malignant diagnosis.

### 3.2. Histopathological Results

Pathology reports revealed malignant disease for 323 patients and benign conditions for 192 patients. The most common malignant pathology was squamous cell carcinoma, of the unkeratinized type, found in 200 patients (57.1%). Keratinized squamous cell carcinoma was identified in 68 patients (19.4%). Other malignant pathologies included rare cancers in 27 patients (7.7%) and non-Hodgkin lymphoma in 24 patients (6.9%). Among the benign conditions, reactive lymphoid hyperplasia was the most prevalent, occurring in 76 patients (46.1%). Other frequent benign pathologies were chronic inflammation in 21 patients (12.7%) and chronic hypertrophic tonsillitis in 15 patients (9.1%). The histopathological results are summarized in Table 3.

### 3.3. Survival Analysis

There is a tendency for mortality rate to rise as N staging increases. Specifically, patients with N3 staging had a 2.87-fold higher risk of mortality compared to patients with N0 staging (*p* = 0.015). In a multivariable analysis, after adjustment for other variables, this association was no longer significant (*p* = 0.389). The present study showed that patients presented a grading of dysplasia of G2 ((71.9%), G3 (12.3%), G1 (2.3%), and in situ (2.6%), as shown in Table 4. In the multivariable analysis, patients with high-grade tumors (G3) had a substantially lower risk of death than those with low-grade tumors (G1), with an OR of 0.12 (*p* = 0.044). In the multivariable analysis, patients with rhinopharyngeal tumors presented a substantially lower risk of death than those with tumors in the hypopharynx, with an OR of 0.14 (*p* = 0.129). In the multivariable analysis, patients with acute respiratory failure had a higher risk of mortality, with an OR of 1.90 (*p* = 0.271). With a *p*-value of 0.048 in the univariable analysis, however, this association was only close to significance.

**Figure 2:**
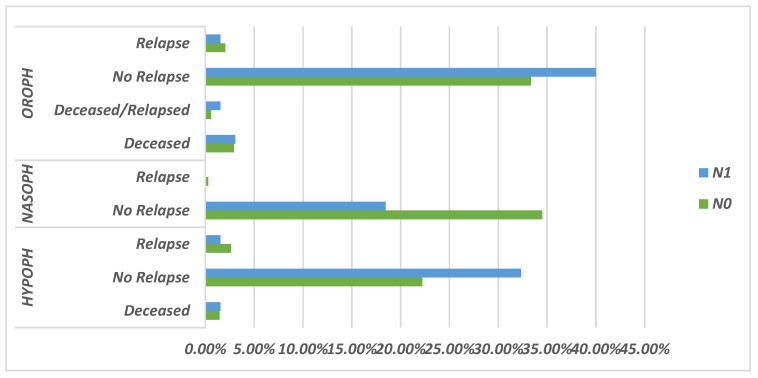
Schematic representation of the prognosis rate based on N0/N1 stage.

As shown in Figure 2, survival time was calculated from initiation of therapy to disease remission. Patients with oropharyngeal tumors had the longest median survival of over 3 years, while median survival could not be estimated for rhinopharyngeal and hypopharyngeal tumors due to insufficient events. However, the survival curve for hypopharyngeal tumors was below that of oropharyngeal tumors, indicating a shorter survival rate.

**Figure 3:**
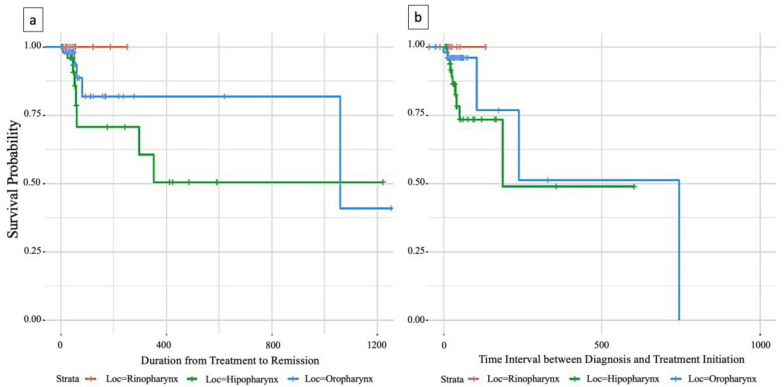
(**a**) Survival analysis of time to therapy initiation and remission in patients with pharyngeal carcinoma; (**b**) time Interval between diagnosis and treatment initiation.

Figure 3a,b present Kaplan–Meier survival curves for 124 patients with N0 and N1 nodal staging, stratified by pharyngeal tumor location.

In the updated analysis presented in Figure 3, survival time was measured from diagnosis to initiation of therapy. Median survival for hypopharyngeal tumors could now be estimated at 186 days, confirming a shorter survival rate compared to oropharyngeal tumors. For oropharyngeal tumors, the updated median survival was 745 days, although the confidence interval was wider due to fewer events.

Nevertheless, both Figure 2 and Figure 3 demonstrate clear regional differences in survival outcomes for pharyngeal cancer patients with N0/N1 staging. Oropharyngeal tumors were associated with the most favorable survival rate, while hypopharyngeal tumors had the worst survival rate. Rhinopharyngeal tumors could not be adequately assessed due to the limited number of cases.

## 4. Discussion

Romania has one of the lowest life expectancy rates in the EU and, despite improvements since 2000, it is still more than six years below the EU average. Similar to other developing countries in the EU, Romania needs to make progress in a number of sectors related to public health [16]. Usually, HNC is a cancer developed by adults with a median age at diagnosis above 50 years, as follows: 66 years for HPV-negative HNSCC, 53 years for HPV-positive HNC, and 50 years for EBV- HNC positive [2,17]. Our findings revealed that patients with cancer were older, with a mean age of 59.7 (9.8) years versus 47.3 (15.6) years in the benign group (*p* = 0.001). If a complete medical examination is not fulfilled, certain symptomatology may be missed; therefore, a delay in the indication for biopsy may cause a delay in diagnosis and treatment. The classic symptoms of HNC depend on both the anatomical site of the primary tumor and the etiology of the tumor. Several aspects of the targeted physical examination raise the possibility of cancer, including i) a 1.5 cm nontender neck mass that is anchored to surrounding tissues and has a skin ulcer over it [18]; ii) the patient’s voice (hoarseness); iii) skin lesions, ulcerations, and asymmetry of head/face/ears; iii) for the oral cavity, trismus and/or limited tongue mobility, any ulcers or masses; and iv) for the pharynx, tonsillar and/or soft palate asymmetry. All these aspects should be assessed during the initial physical examination. Furthermore, bimanual palpation of the mouth’s floor is indicated to check for any induration. Because occult primary tumors may develop from anatomic sites such as the nasopharynx, base of the tongue and hypopharynx that are not otherwise easily examined, additional fiberoptic evaluation of the aerodigestive sites should be performed [19,20]. The main curative therapy strategies for locally or locoregionally HNC are resection, radiotherapy, and systemic therapy. Surgery is commonly used for cancers of the oral cavity, whereas radiation may be more commonly employed for pharyngeal cancers [21]. However, the treatment strategy should prioritize preservation of function while pursuing a curative approach. Resection or radiation can cure approximately 80% of patients with tiny primary cancers that have only one or no clinically significant nodes involved, yet a significant proportion of these patients have N0–N1 stage malignant histopathological diagnosis.

Nodal levels require the most attention during treatment planning and post-treatment surveillance periods. Our hope is that patients, surgeons, and radiation therapists will use these data to decide whether to electively treat the neck in the N0-N1 setting and, if so, where the treatment should be focused. Therefore, a benefit– risk assessment can be made to determine whether the neck should be treated or observed as part of initial cancer therapy. These data demonstrate the feasibility of FDG-PET/CT in guiding the personalization of neck management for cN0-staged HNC [22], although certain forms of cancer were classified as N0 at hospital admission, yet 21.8% of squamous cell carcinomas relapsed after oncological treatment. On the other hand, despite numerous attempts to create preventative services over the years, they are scarcer, harder to obtain, and are only partially or even not covered by health insurance or government programs. At the time of diagnosis, more than 50% of patients with squamous cell carcinoma of the head and neck have local lymph node involvement [23]. The prognosis of people with squamous cell carcinoma of the upper aerodigestive tract is largely based on the status of their cervical lymph nodes. Therapy failures in head and neck cancer are considered to be critical—a challenging situation that plays a significant role in morbidity and mortality [24]. Currently, computed tomography (CT) is the main diagnostic technique for N0 staging. Other techniques include magnetic resonance imaging (MRI), ultrasonography (US), and, in some circumstances, positron emission tomography-computed tomography (PET-CT). However, it is estimated that 20–30% of cases do not present clinically evident lymph node metastases [25,26]. Regarding early diagnosis, the fiberoptic evaluation of the aerodigestive sites is available in most outpatient specialty clinics and hospitals and is sometimes even covered by insurance; however, providers are usually located in big cities, and patients from rural or disadvantaged areas have limited access due to geographic, informational, and occasionally financial barriers.

TNM/AJCC staging plays an essential role in tumor treatment planning and prognostic assessment. The rate of lymph node and distant metastases of pharyngeal cancer is increased; the rate of lymph node metastasis at first presentation is high with a distribution of N3 17.4%, N2 10.3%, and N1 15.4%, the rate of possible metastatic lymph node presence at first presentation of patients is 33%. The presence of a single metastatic lymph node (LN) commits patients to an advanced-stage disease category and has been shown to confer up to a 50% decrease in overall survival (OS). The control rate for primary and cervical lymph node metastases has gradually improved in patients with squamous cell carcinoma (SCC), showing improved treatments. Therefore, identifying the high-risk patient presenting distant metastasis represents a key factor to ensure effective treatment as soon as possible. The incidence of uncontrolled primary tumor and cervical lymph node metastases may lead to distant metastases during the development of these recurrent tumors [27,28].

Even after treatment, 30–60% of patients diagnosed at an advanced stage with successful remission will develop recurrent locoregional cancer or second primary cancer. Therefore, the number of affected lymph nodes of a cancer patient may be a better measure of prognosis [5,29]. Due to the combinations of treatments, they all have their specific outcome, including physical, emotional, functional and social sequelae, and occupational dysfunction, as well as a profound effect on the families of patients with HNC. In the past few years, the effect on a cancer patient’s quality of life (QoL) has gained major importance. In our study, most patients had a biopsy; however, 9.7% of these patients presented acute respiratory failure (*p* < 0.001), while we had to perform emergency tracheotomy in 6.3% of cases and total laryngectomy in 0.9% of cases.

Histologically, the progression to mild, moderate, and severe carcinoma in situ and, finally, invasive HNC carcinoma follows an ordered series of steps, beginning with epithelial cell hyperplasia, followed by dysplasia [30].

Depending on the location of the primary tumor, patients with HNC may experience specific symptoms, such as oral dysfunction and swallowing or speech problems, during treatment, which may often improve 6 months post-treatment [31,32]. The early stages of head and neck cancer can be cured via surgery or radiotherapy, with promising results; however, certain risk factors may be involved in disease recurrence. In the era of organ preservation, practitioners are leaning more toward the concurrent use of radiotherapy and chemotherapy instead of directly opting for surgery [33].

Currently, there is no standard or routine screening test to diagnose oral and pharyngeal cancers, and no screening guidelines have been provided for the early detection of oral and pharyngeal leukoplakia and erythroplakia lesions or cancers in the general population. There is also the problem of the absence of a cancer program separate from the general medical history, especially for cancer patients from private institutions and which should handle the treatment, history, stage of a patient, and tertiary prevention. Nevertheless, for better assessment of the patient, all institutions providing healthcare in the area of cancer must work together to develop a well-organized regimen for better outcomes and easy follow-up of the patient. Clinicians gathering past medical histories should also ask patients about tobacco use and alcohol consumption in order to assess whether smoking cessation intervention and/or counseling (for tobacco) is needed. Future research is needed to understand the risk factors and impact of financial toxicity on HNC patients, as well as the effectiveness of interventions, such as financial navigators, policies addressing drug price transparency, and value-based insurance design, in alleviating the cancer-related financial burden. What we must strive to understand when assessing the influence of financial burden on HNC care is the patient-level impact of perceived financial harm on clinically meaningful outcomes.

Though the many strengths of this study, our findings are representative of patients treated in a single healthcare system with similar treatment-related costs and relatively homogenous demographics, which may not be representative or generalizable to all HNC patients. Furthermore, despite our high participation rate, future studies should be multi-institutional in nature, including analyses of additional financial coping mechanisms, and should also consider larger sample sizes to increase the generalizability of findings to allow subgroup analysis in regression modeling. In addition, it should be noted that comorbidities and ethnicity were not available and may represent unidentified sources of confounding.

## 5. Conclusions

In conclusion, this study highlights the importance of identifying risk factors associated with malignancy and toxicity in patients with head and neck cancer. Early diagnosis and access to multidisciplinary teams are crucial to improving survival rates and health-related QoL. Financial limitation remains a major concern for patients with HNC, and further research is needed to understand the impact and efficacy of interventions. The absence of routine screening tests and screening guidelines for oral and pharyngeal cancers represents a significant barrier to the secondary prevention of HNC. Despite the strengths of this study, larger multi-institutional studies are needed to increase generalizability and account for potential confounders, such as comorbidities and ethnicity. Overall, our findings highlight the need for a well-organized and coordinated approach to HNC care that addresses the various challenges and barriers faced by patients and healthcare providers.

## Figures and Tables

**Table 1:** Romania compared to worldwide incidence.

Type of Cancer	Romania				Worldwide Annual Number of New Cancer Cases (%)
	New Cases (No)	Rank by Incidence Worldwide	Percentage of Death (%)	5-Year Survival Prevalence/100,000	
Nasopharynx	403	27th	0.34	6.66	0.5
Hypopharynx	634	24th	0.22	5.76	0.4
Oropharynx	1351	21st	1.4	19.12	0.7

**Table 2:** Comparison of clinical characteristics and outcomes between benign and malignant cases.

	Benign (*n* = 165)	Malign (*n* = 350)	Total (*n* = 513)	*p*-Value
Age				<0.001 ^1^
Mean (SD)	47.3 (15.6)	59.7 (9.8)	55.7 (13.3)	
Range	17.0–83.0	21.0–86.0	17.0–86.0	
Site				<0.001 ^2^
Hypopharynx	7.0 (4.2%)	150.0 (42.9%)	157.0 (30.5%)	
Oropharynx	55.0 (33.3%)	144.0 (41.1%)	199.0 (38.6%)	
Rhino pharynx	103.0 (62.4%)	56.0 (16.0%)	159.0 (30.9%)	
Sex				<0.001 ^2^
M	88.0 (53.3%)	302.0 (86.3%)	390.0 (75.7%)	
F	77.0 (46.7%)	48.0 (13.7%)	125.0 (24.3%)	
Year				0.739 ^2^
2014	22.0 (13.3%)	57.0 (16.3%)	79.0 (15.3%)	
2015	29.0 (17.6%)	61.0 (17.4%)	90.0 (17.5%)	
2016	41.0 (24.8%)	83.0 (23.7%)	124.0 (24.1%)	
2017	33.0 (20.0%)	79.0 (22.6%)	112.0 (21.7%)	
2018	40.0 (24.2%)	70.0 (20.0%)	110.0 (21.4%)	
Area				0.466 ^2^
Urban area	91.0 (55.2%)	181.0 (51.7%)	272.0 (52.8%)	
Rural area	74.0 (44.8%)	169.0 (48.3%)	243.0 (47.2%)	
Nodal staging				<0.001 ^2^
N0	146.0 (88.5%)	199.0 (56.9%)	345.0 (67.0%)	
N1	10.0 (6.1%)	54.0 (15.4%)	64.0 (12.4%)	
N2	4.0 (2.4%)	36.0 (10.3%)	40.0 (7.8%)	
N3	5.0 (3.0%)	61.0 (17.4%)	66.0 (12.8%)	
Days of admission				<0.001 ^1^
Mean (SD)	3.7 (2.7)	5.4 (4.7)	4.9 (4.2)	
Range	1.0–23.0	1.0–37.0	1.0–37.0	
Deceased	1.0 (0.6%)	33.0 (9.4%)	34.0 (6.6%)	<0.001 ^2^
Acute respiratory failure	0.0 (0.0%)	34.0 (9.7%)	34.0 (6.6%)	<0.001 ^2^

Legend: ^1^ linear model ANOVA; ^2^ Pearson’s chi-squared test.

**Table 3:** Histopathological diagnosis.

Histopathological Diagnosis	(*n* = 515)
Benign	*n* = 165
Inflammatory polyp	3.0 (1.8%)
Adenoid vegetations	2.0 (1.2%)
Chronic hypertrophic adenoiditis	5.0 (3.0%)
Chronic hypertrophic tonsillitis	15.0 (9.1%)
Chronic granulomatous inflammation	6.0 (3.6%)
Reactive hyperplasia of lymphoid follicles	76.0 (46.1%)
Squamous papilloma	11.0 (6.7%)
Chronic inflammatory process	21.0 (12.7%)
Malign	*n* = 350
Squamous cell carcinoma	200.0 (57.1%)
Keratinizing squamous cell carcinoma	68.0 (19.4%)
In situ squamous cell carcinoma	7.0 (2.0%)
UCNT	24.0 (6.9%)
NHL	24.0 (6.9%)
Other cancers	27.0 (7.7%)

Legend: UCNT—undifferentiated carcinoma of the nasopharynx; NHL-non-Hodgkin lymphoma.

**Table 4:** Univariate and multivariate odds ratio (OR) for overall survival.

Dependent: Deceased		No	Yes	OR (Univariable)	OR (Multivariable)
N staging	N0	327 (94.8)	18 (5.2)	-	-
	N1	61 (95.3)	3 (4.7)	0.89 (0.20–2.74, *p* = 0.860)	0.41 (0.06–1.62, *p* = 0.266)
	N2	36 (90.0)	4 (10.0)	2.02 (0.56–5.77, *p* = 0.226)	1.43 (0.34–4.86, *p* = 0.592)
	N3	57 (86.4)	9 (13.6)	2.87 (1.18–6.56, *p* = 0.015)	1.56 (0.55–4.16, *p* = 0.389)
Grading	No	31 (91.2)	3 (8.8)	-	-
	G1	6 (85.7)	1 (14.3)	1.72 (0.08–16.37, *p* = 0.661)	0.51 (0.02–7.31, *p* = 0.641)
	G2	206 (91.2)	20 (8.8)	1.00 (0.32–4.43, *p* = 0.996)	0.17 (0.03–1.11, *p* = 0.051)
	G3	35 (92.1)	3 (7.9)	0.89 (0.15–5.09, *p* = 0.887)	0.12 (0.01–0.95, *p* = 0.044)
	In situ	5 (62.5)	3 (37.5)	6.20 (0.93–43.32, *p* = 0.049)	1.13 (0.11–11.70, *p* = 0.916)
Subsite	Hypopharynx	142 (90.4)	15 (9.6)	-	-
	Oropharynx	181 (91.0)	18 (9.0)	0.94 (0.46–1.96, *p* = 0.869)	1.23 (0.52–2.95, *p* = 0.631)
	Rhino pharynx	158 (99.4)	1 (0.6)	0.06 (0.00–0.30, *p* = 0.007)	0.14 (0.01–1.27, *p* = 0.129)
Acute respiratory failure	No	452 (94.0)	29 (6.0)	-	-
	Yes	29 (85.3)	5 (14.7)	2.69 (0.87–6.95, *p* = 0.048)	1.90 (0.55–5.69, *p* = 0.271)

## Data Availability

All raw data used in the current study can be found in the archives of the Timișoara Municipal Emergency Clinical Hospital, Department of Otorhinolaryngology.
